# Validation of the VALVEX Questionnaire for Assessing Patient Experience During the Transcatheter Aortic Valve Implantation Process

**DOI:** 10.3390/jcm14238562

**Published:** 2025-12-02

**Authors:** Miryam González-Cebrian, Elena Calvo, Sara Alonso Meléndez, Héctor Gómez García, Francisco Javier Rubio Gil, Ignacio Cruz-González, Pedro L. Sánchez, Luis Miguel Rincón, José Luis Mendoza-García

**Affiliations:** 1 Department of Cardiology, University Hospital of Salamanca, 37007 Salamanca, Spain; salonsome@saludcastillayleon.es (S.A.M.); hgomezg@saludcastillayleon.es (H.G.G.); fjrubio@saludcastillayleon.es (F.J.R.G.); cruzgonzalez.ignacio@gmail.com (I.C.-G.); plsanchez@saludcastillayleon.es (P.L.S.); lmrincond@saludcastillayleon.es (L.M.R.); 2 Instituto de Investigación Biomédica de Salamanca (IBSAL), 37007 Salamanca, Spain; 3 Department of Nursing, Universidad de Salamanca (USAL), 37008 Salamanca, Spain; 4 Department of Medicine, Universidad de Salamanca (USAL), 37008 Salamanca, Spain; 5 CIBER-CV Instituto de Salud Carlos III (ISCIII), 28029 Madrid, Spain; elenacb@gmail.com; 6 Department of Cardiology, University Hospital of Bellvitge, 08907 Barcelona, Spain; 7 GRIN-IDIBELL, 08036 Barcelona, Spain; 8 Servicio de Medicina Preventiva, University Hospital of Torrevieja, 03186 Alicante, Spain; mendoza_josgar@gva.es

**Keywords:** aortic valve stenosis, transcatheter aortic valve replacement, patient experience, patient-reported experience measures (PREMs), questionnaires, health care quality assessment

## Abstract

**Background and Objectives:** Degenerative aortic stenosis has become the most common primary valve lesion referred for intervention, with thousands of transcatheter aortic valve implantation (TAVI) procedures being performed annually. To date, there are no quantitative tools capable of evaluating patient experience adapted to percutaneous procedures of this nature. The goal of this study is to propose and validate the VALVEX questionnaire as the reference tool to assess patient experience throughout the TAVI process and to identify areas for improvement in healthcare. **Methods:** This was a cross-sectional observational study with a 30-day follow-up that included consecutive patients > 18 years of age undergoing elective TAVI in two Spanish hospitals. A multidisciplinary panel including patients was constituted to design, refine, and pilot the VALVEX questionnaire. During its completion 30 days after the procedure, the dimensions assessed were the care provided before, during, and after TAVI, as well as the facilities and overall patient satisfaction. Reliability, validity, and feasibility were analysed during the validation phase. **Results:** A total of 335 patients were enrolled and filled out the VALVEX questionnaire (mean age 81.1 ± 5 years, 52% female). Evaluation of feasibility showed a mean completion time of 7.8 ± 2.3 min, with a 2.1% rate of unanswered questions. Cronbach’s alpha index was 0.912, with high internal reliability measurements for all the assessed dimensions. An intraclass correlation coefficient (ICC) of 0.851 was determined as an indicator of stability. The Delphi panel achieved a consensus of over 85% in clarity, coherence, relevance, and sufficiency. **Conclusions:** We have successfully validated the VALVEX questionnaire as the first quantitative tool specifically designed to measure and evaluate patient experience throughout the TAVI procedure and recovery. This questionnaire is a valid, reliable, simple, and easy-to-use resource that could be used as a reference tool to assess patient experience for TAVI or other percutaneous cardiac interventions.

## 1. Introduction

Aortic stenosis (AS) is the most common primary valvular disease referred for intervention. Its prevalence is rapidly increasing, in part, because of the ageing population and increased life expectancy [1,2,3,4].

Transcatheter aortic valve implantation (TAVI) has become a standard therapy for the treatment of severe AS, with approximately 100,000 procedures performed annually in Europe. It is considered the treatment of choice for patients at high surgical risk, and a valid alternative to surgical aortic valve replacement in patients at intermediate or low surgical risk [5,6,7,8,9,10].

Clinical outcomes after TAVI are clearly established and monitored, including mortality, need for pacemaker implantation or readmissions [11]. On the other hand, there is a lack of standardized methods for evaluation of patient experience in the general context of percutaneous interventions, and TAVI in particular [12]. Some qualitative scales have been proposed for their application in different phases of the patient journey, but no quantitative tools are used due to the lack of specific scales for this process [13,14,15,16,17].

While there are general experience surveys for hospitalized patients, such as the Picker Patient Experience Questionnaire PPE-15 [18], and other cardiology-specific surveys that assess the impact on quality of life and take into account patient expectations, all of them are clearly out of the scope of these patients [19,20,21].

There are some distinctive features of the patient undergoing TAVI—a percutaneous procedure with rapid recovery and usually short hospital stay—in addition to other factors, that make the aforementioned questionnaires unsuitable for reflecting the experience of a TAVI patient.

Furthermore, the official document of the Spanish Ministry of Health (Cardiovascular Health Strategy ESCAV) [22], in its “knowledge management, research, and innovation” axis, establishes that “patient-reported outcomes and experiences must be incorporated into the institutional results report” and speaks of “the value-based healthcare methodology” (PROM patient reported outcomes measure, PREM patient reported experience measure) as an action to be promoted in cardiovascular health. Along these lines, the Spanish Society of Cardiology (SEC), with the PROMCAR project [23], also promotes an explicit initiative that proposes that patient experience should play a role in the evaluation. The European Society of Cardiology (ESC) and European Association for Cardio-Thoracic Surgery (EACTS) guidelines [24] recommend establishing a dedicated point of contact to address these concerns and facilitate a shared decision between the Heart Team and the informed patient and relatives. In the same line as guidelines, there are other initiatives proposing that patient experience should play a central role in this patient-centred process [25].

To overcome this unmet need, the VALVEX questionnaire has been designed as a targeted questionnaire focused on patient experience for those undergoing TAVI (Table A1). While its development has been recently published [26], validation of this questionnaire is still pending. To ensure methodological quality, it is essential that the results of the questionnaire can be correctly interpreted, which makes a full validation of the instrument necessary in a larger and more heterogeneous cohort, evaluating Its psychometric properties [27,28,29,30,31,32].

The goal of this study is to propose the VALVEX questionnaire as the reference tool to assess patient experience during the TAVI procedure by its first validation in a cohort of patients undergoing this procedure, and to identify areas for improvement in healthcare.

## 2. Materials and Methods

### 2.1. Study Design

This was a cross-sectional observational study to validate the VALVEX questionnaire, previously designed to evaluate the experience of patients undergoing TAVI. The study was conducted at two large tertiary centres in Spain (Complejo Asistencial Universitario de Salamanca and Hospital Universitario de Bellvitge, Barcelona) between December 2023 and January 2025, in two phases: a previously published pilot phase (*n* = 100) and final validation phase (*n* = 235).

### 2.2. Population and Sample

Consecutive patients with severe AS undergoing TAVI. The inclusion criteria, which did not differ between the pilot and validation samples, were age ≥ 18 years, ability to understand and respond to the questionnaire, and informed consent. The only exclusion criteria were refusal to participate or physical or cognitive limitations that prevented from completing the questionnaire, even with assistance.

### 2.3. Questionnaire Development

The VALVEX questionnaire was previously designed based on a literature review in Medline, PubMed, and SciELO databases (November 2022–March 2023) and a content analysis, from which an initial pool of 200 questions was generated. A multidisciplinary panel was constituted with 12 experts (four cardiologists, seven nurses with TAVI experience, and three experts in Quality of Care and Patient Safety), and eight patients who had previously undergone a TAVI procedure. An iterative process of semantic and comprehension reduction and improvement was performed using the Delphi technique (3 rounds) until achieving the final version. The resulting VALVEX questionnaire is composed of 29 items grouped into four dimensions: pre-procedure, intra-procedure, environment and facilities, and post-procedure/follow-up (Table 1). Each item is rated using a five-point Likert scale, from “Strongly disagree” to “Strongly agree”. The complete questionnaire is included as Table A1.

### 2.4. Data Collection

The questionnaire was provided at the nursing follow-up consultation 30 days after the TAVI procedure. Completion was carried out predominantly through a structured interview conducted by nursing staff. Only in isolated cases (1–2 patients) was self-administration allowed, due to individual circumstances that did not affect the overall data collection procedure. They were then digitized into an Excel database and analysed.

### 2.5. Questionnaire Validation

The questionnaire’s validation included an analysis of its psychometric properties: reliability, validity, and feasibility.

Reliability: Internal consistency was assessed using Cronbach’s alpha coefficient, with values from 0.70 to 0.79 considered acceptable, 0.80 to 0.89 considered good, and ≥0.90 considered excellent. Temporal stability was determined using the intraclass correlation coefficient (ICC), with values above 0.6 considered adequate.

Validity: Content validity was established using the Delphi technique, in which a panel of experts evaluated the items for clarity, coherence, relevance, and adequacy. Construct validity was assessed using the cluster method, comparing the means of the dimensions between the two phases of the study using the Student *t*-test for independent samples with a significance level of *p* < 0.05.

Feasibility: Feasibility was determined based on three indicators: mean completion time, percentage of unanswered items, and degree of acceptance. It was considered feasible if the questionnaire could be completed in <10 min, with <5% of unanswered items, and with greater than 80% of patients reporting ease of understanding. Furthermore, the professionals’ assessment of the practical usefulness of the questionnaire was collected.

### 2.6. Statistical Analysis

A descriptive analysis of the sample’s sociodemographic and clinical variables was performed using measures of central tendency (mean and median) and dispersion (standard deviation and interquartile range) for quantitative variables, as well as frequencies and percentages for categorical variables. The psychometric properties of the VALVEX questionnaire were subsequently evaluated. In all cases, a statistical significance level of *p* < 0.05 was adopted. The sample size was estimated following commonly accepted methodological criteria for questionnaire validation studies, which recommend including between 5 and 10 participants per item to ensure the stability of the psychometric estimates and the adequacy of the factor analysis [33]. Given that the VALVEX questionnaire established 29 items, the required sample size was estimated between 145 and 290 subjects. Data analysis was performed using SPSS version 23.

### 2.7. Ethics

The study follows the ethical considerations of the Declaration of Helsinki. Ethics Committee at both sites approved the study protocol (ID code PI 2023 12 1468) and all patients signed the informed consent form prior to study evaluations.

During the entire data collection process, confidentiality was maintained in accordance with the provisions established by the National Organic Law 3/2018 on the Protection of Personal Data and Guarantee of Human Rights as well as the European Regulation 2016/679 for General Data Protection Regulation.

## 3. Results

A total of 335 questionnaires were analysed (phase 2023: *n* = 100; phase 2024: *n* = 235). No significant differences were observed between phases regarding age (mean 81 years in both groups), sex, or educational level. No significant differences were observed between phases regarding cognitive impairment. The inclusion of these variables allows for the assessment of their potential influence on questionnaire completion, although the high rate of fully completed responses (94%) suggests that neither educational level nor cognitive function meaningfully affected patients’ ability to complete the instrument (Table 2).

Final structure of the instrument: The final version of the questionnaire consisted of 29 items grouped into four dimensions, with Likert-scale responses ranging from 1 (strongly disagree) to 5 (strongly agree).

Feasibility: The mean questionnaire completion time was 7.8 ± 2.3 min. The overall unanswered item rate was 2.1%, below the accepted 5% threshold. Ninety-four percent of patients responded to all items, 97% found them easy to understand, while 96% of expert professionals positively rated the items’ clarity, relevance, and adequacy. Since only 1–2 patients completed the questionnaire in a self-administered manner, it was not possible to perform statistical comparisons between modes of administration.

Table 3 shows the item distribution across both phases of the study. Overall, a high proportion of responses in the ‘agree’ and ‘strongly agree’ categories was observed across all items and dimensions evaluated.

In the Pre-Procedure Care dimension, more than 85% of patients in both phases stated they agreed or strongly agreed with items Q1 to Q5, with item Q5 ‘I am satisfied with the information received by the nursing staff’ standing out, with 72.6% and 72.3% responding ‘strongly agree’ in 2023 and 2024, respectively.

In the Procedure-Centred Care and Admission dimension, items Q8 to Q14 reflected a similar pattern, with over 90% of responses in the ‘agree’ or ‘strongly agree’ categories. Item Q10 ‘I am satisfied with the information the doctors gave me’ reached 68.0% and 69.4% ‘strongly agree’ scores in each phase.

In the Infrastructure dimension, (Q15 to Q19) also presented a positive perception. In particular, item Q15 ‘I am satisfied with the cleanliness of the facilities’ was rated as ‘strongly agree’ by 55.0% of patients in 2023 and 60.0% in 2024.

In the Post-procedure Focused Care dimension, items Q20 to Q27 showed high satisfaction, especially item Q27 ‘I would recommend that other patients in my situation undergo the TAVI procedure’, which received 70.5% and 71.9% ’strongly agree’ responses in the first and second phases, respectively.

Table 4 details the overall distribution (*n* = 335), showing a clear trend toward higher satisfaction categories in all dimensions of the questionnaire.

Internal reliability: The questionnaire showed an excellent internal consistency, with an overall Cronbach’s alpha of 0.912. The coefficients by phase and overall are summarized in Table 5. Cronbach’s alpha coefficient for each dimension was: Pre-procedure: 0.713; Intra-procedure: 0.893; Infrastructure/Facilities: 0.776; and Post-procedure/Follow-up: 0.787. In both phases of the study, all values exceeded the accepted threshold of 0.70, demonstrating the instrument’s excellent reliability for assessing the experience of patients undergoing TAVI. The intra-procedure dimension showed the highest internal consistency.

Temporal stability, assessed by test–retest, showed an intraclass correlation coefficient (ICC) for individual measures of 0.851 (95% CI: 0.159–0.955; *p* < 0.001), indicating adequate temporal reliability.

Validity: Regarding content validity, the Delphi panel achieved a consensus of over 85% according to all the established judgement categories: clarity, coherence, relevance, and sufficiency. As for construct validity, no statistically significant differences were observed between the two phases (*p* > 0.05), suggesting stability of the questionnaire structure.

Comparison between phases: When comparing the pilot phase (*n* = 100) and the validation phase (*n* = 235), improvements were observed in Cronbach’s alpha coefficients, especially in the pre-procedure dimension (from 0.695 to 0.723), which may reflect greater internal homogeneity following item optimization.

## 4. Discussion

This study presents the validation of the first quantitative questionnaire used to specifically measure and evaluate the experience of patients undergoing TAVI. We have proved that the VALVEX questionnaire is a valid, reliable, simple, and easy-to-use tool. Based on the obtained results, we can propose it as a reference tool to assess patient experience for those undergoing TAVI or other similar percutaneous structural heart interventions.

Assessment of patient experience emerges as the ideal tool for placing the patient at the centre of care, facilitating their active participation in the healthcare system. It also provides useful information for assessing the organization’s culture and for evaluating, designing, and improving healthcare processes so that they respond, as far as possible, to the needs, circumstances, and environment of patients [34,35].

For this reason, patient experience has begun to be measured with instruments administered to the patients themselves. These inexpensive tools can reach a large number of patients and offer relatively simple analysis, enabling improvements in patient-centred care and conveying patient feedback [36].

Although VALVEX is defined as a PREM, two items in the post-procedural domain (P21 and P22) capture the subjective perception of recovery, as patients identified these aspects as essential components of their care experience during the qualitative development phase. These items do not measure clinical outcomes, but experiential perceptions that influence the overall evaluation of the care process, in line with contemporary conceptual frameworks of patient experience.

Navarro et al. [37], in their systematic review, described that assessing the patient experience is associated with improved physical and mental status, as well as lower frequency of visits to the emergency department, hospitalizations, and length of hospital stay, in addition to other favourable outcomes.

Regarding the TAVI procedure, the core of our study, analysing patient satisfaction and experience with the TAVI process shows that it improves therapeutic adherence, provides continuity of care, improves care processes, and even reduces hospital stay, as well as readmissions and visits to the emergency department after discharge [12]. However, few studies on TAVI measure and evaluate the patient experience by providing scientific evidence. This could be related to the lack of specific questionnaires for this purpose.

Some patients are referred for TAVI in an acute setting (e.g., hospitalization for decompensated heart failure). Although the questionnaire has been tested and validated in patients with scheduled implantation, its use can be generalized since the evaluation is performed one month after implantation [38].

Therefore, based on the lack of any instrument, validated or unvalidated, we believe the existence of a specific questionnaire for patients undergoing TAVI is necessary. The VALVEX questionnaire was initially designed and implemented in a pilot study with 100 patients, which showed high satisfaction and acceptable internal consistency. The results were recently published [26].

The development and validation of a questionnaire is relatively complex and requires an appropriate methodology to ensure its validity and reliability [27,28,29]. The literature shows consensus that these two metrics are essential for assessing the accuracy of any self-designed questionnaire [27].

As reflected in the literature [27,28,29,30,31,32] the intended measurement must be clearly defined (construct definition and validity) and must be appropriate for the health problem under study. During the development of this questionnaire, a multidisciplinary group was created to design it, starting with an exhaustive review of the literature. We identified the absence of a specific scale for measuring patient experience during the TAVI process. This fact served as a catalyst for continuing the study and developing and validating a questionnaire for this purpose. We also defined the target population, as well as the questionnaire format, delivery methodology, and completion time—all of which are relevant data for question formulation and writing.

The Delphi method is widely used as a consensus method among experts in questionnaire validation, with opinions obtained through this technique being considered more consistent than individual ones. Furthermore, it is a very useful tool for designing and validating new instruments when none exists that meet the needs of the research being conducted [39,40]. Therefore, we used this technique to develop and validate a new questionnaire that would allow us to understand patient experience and satisfaction with the TAVI process, thus enabling continuous improvement of care and quality of care.

Inclusion of patients in healthcare processes, and establishing patient-centred care models leads to better health outcomes and increased patient satisfaction and experience throughout their disease process [21,41]. Furthermore, when developing and designing experience and satisfaction questionnaires, patients should be part of multidisciplinary teams and expert groups, as it is essential to include their voice and perspective, which adds value to our questionnaire [41].

Like other questionnaires measuring patient experience, our questionnaire has the advantage of a small number of items and can be completed in a short time [18,42]. The use of a 1 to 5 response scale is simple and intuitive for the patient.

In contrast to other studies, our questionnaire considered organizational, environmental, and infrastructure issues in developing our questionnaire, as these also influence the patient experience [18,19,20,21,43].

Questionnaires focused on patient experience in cardiology are not directed at the TAVI process or patient; they focus on measuring patient satisfaction than the experience itself, and most assess the patient only in the post-discharge phase [19,20,21]. The VALVEX questionnaire provides an evaluation of the experience that encompasses the entire care process, as it covers all aspects of care, from diagnosis to the procedure, admission, and follow-up after discharge. Unlike other questionnaires, it is divided into four distinct dimensions.

The fact that the questionnaire was completed one month after the TAVI procedure, during the follow-up nursing consultation, allowed the patient to easily assess and remember all the steps, avoiding forgetfulness or differing interpretations if the evaluation is made long after the procedure.

Having participated in the design, both patients and professionals from different hospitals and geographical areas make the VALVEX questionnaire a useful tool for benchmarking between centres.

VALVEX demonstrated high feasibility, reflecting that this questionnaire is easy for patients to understand and complete. This is especially interesting given the elder age of the study participants, with an overall rate of unanswered items of 2.1%. The acceptance rate of 97% by patients and 96% by professionals reflects the quality of the questionnaire.

The incorporation of educational level and cognitive impairment in the characterization of the sample allows for contextualization of the questionnaire’s applicability. Despite the advanced age of the studied population, the high completion and comprehension rates demonstrate that the questionnaire is appropriate even for patients with low educational levels or mild cognitive impairment.

Regarding reliability, the questionnaire shows great consistency both in the pilot phase and in the second phase of validation. Although all items passed the reliability tests (Cronbach’s alpha > 0.70), the items that contributed most to overall consistency were those corresponding to the intraprocedural phase (almost reaching an excellent level of reliability), while those in the preprocedural dimension showed somewhat lower consistency. This finding could be due to the heterogeneity in the perception of the information received in the initial phase or to a lesser emotional impact on the patient at this stage. This highlights the need to reinforce the guidelines outlined in the new ESC/EACTS 2025 guidelines for the management of valvular heart disease, which emphasize the need to maintain systematic Heart Team meetings, clinical governance, and shared decision-making with the patient throughout the process [24].

The analysis of temporal stability showed good to excellent reliability, confirming that the instrument maintains its psychometric properties upon repeated administrations. However, this analysis was performed in a limited group of patients, so it should be confirmed in larger multicentre studies to more robustly assess stability.

Although the VALVEX questionnaire was structured based on a predefined conceptual framework using the Delphi technique, literature review, and expert consensus, we acknowledge that a confirmatory factor analysis (CFA) could provide additional evidence regarding its structural validity. The sample size of the present study was not specifically designed to support a robust CFA; therefore, this analysis is proposed as a future research step within the framework of an expanded multicentre validation.

Moreover, the test’s good stability (ICC = 0.92) confirms its usefulness and temporal reproducibility when used by other populations and hospitals. Study population comprised a total of 335 patients, widely above the necessary threshold determined during sample size estimation, which reinforces the robustness of the analyses performed.

We can affirm that the VALVEX questionnaire meets the requirements of a valid questionnaire, being a simple, viable tool accepted by patients and professionals. It is reliable and accurate, with construct and content validity. However, we cannot forget that validation is a continuous and dynamic process, as is the TAVI process. Therefore, in future studies, we must consider its sensitivity to change over time and analyse and measure changes in both the population and the process, adapting our questionnaire accordingly.

Based on the nature of the questionnaire, the VALVEX questionnaire could be useful for measuring patient experience in other percutaneous cardiac interventions with similar technical profile, such as other valve interventions (mitral or tricuspid) or ablation.

### Limitations

This study has some limitations. First, although self-administration of the VALVEX questionnaire was considered, in practice only 1–2 patients used this format. Therefore, it was not possible to analyse differences between modes of administration. Nevertheless, the vast majority of questionnaires were completed with nursing assistance, ensuring homogeneity in data collection.

Second, the VALVEX questionnaire was developed and validated within the Spanish healthcare system, so its applicability to other countries may be influenced by cultural, linguistic, and organizational differences. A transcultural adaptation and international external validation will be required before using it in other healthcare systems.

Moreover, the study was conducted at two centres with extensive experience in TAVI, which may influence the generalization of the results to other healthcare settings. Although the sample size was adequate, stratified random sampling was not used, which could have affected the representation of certain population subgroups. Despite these limitations, the VALVEX questionnaire proved to be a valid, reliable, and feasible tool for assessing the TAVI patient experience, with the potential to improve patient-centred care.

To consolidate the VALVEX questionnaire as a reference tool, a multicentre external validation is necessary to confirm its usefulness in different settings, populations and others percutaneous interventions besides TAVI. Future research should evaluate its sensitivity to change, allowing for the measurement of the impact of interventions focused on improving the patient experience. Exploring its predictive validity in relation to clinical outcomes (such as therapeutic adherence or readmissions) could position it as a proxy indicator of quality of care. Finally, its adaptation to digital formats and integration into electronic medical record systems would facilitate its systematic use in strategies for the evaluation and continuous improvement of TAVI care.

## 5. Conclusions

We have successfully validated the VALVEX questionnaire, the first quantitative tool to evaluate the experience of the patient undergoing the most prevalent heart valve intervention, TAVI. The VALVEX questionnaire demonstrates satisfactory internal consistency, reliability, and reproducibility and can therefore be considered a valid and reliable tool, highly useful for placing the patient at the centre of attention and in the pursuit of improved healthcare and quality of care.

## Figures and Tables

**Table 1:** Dimensions of the VALVEX questionnaire and distribution of items.

Category	Items
Care provided before the TAVI procedure	1. When I was diagnosed with the disease, they explained to me other treatment options (if there were any)
	2. When I was diagnosed with the disease, I was able to take part in the decision to undergo TAVI.
	3. I believe that the decision to undergo the TAVI procedure was the right one.
	4. I am satisfied with the information provided by the medical staff when I was diagnosed with my disease.
	5. I am satisfied with the information provided by the nursing staff when I was diagnosed with my disease.
Care provided during the TAVI procedure and admission	6. I believe it was easy to get to the unit where I had to be admitted.
	7. I believe that the waiting time from diagnosis to treatment (TAVI procedure) was appropriate.
	8. I am satisfied with the care received during the procedure.
	9. I am satisfied with the information the doctors gave me throughout my hospital stay.
	10. I am satisfied with the information the nursing staff in the units provided me throughout my hospital stay.
	11. I am satisfied with the kindness and professionalism with which the healthcare staff treated me.
	12. I believe that the healthcare staff explained the process to me in easy-to-understand language.
	13. I believe that the time spent by the unit nurse was adequate.
	14. I am satisfied with the care provided by the healthcare staff during my stay.
Facilities	15. I am satisfied with the cleanliness of the facilities.
	16. I am satisfied with the noise level in the facilities.
	17. I am satisfied with the meals that were served to me.
	18. I am satisfied with the comfort of the room.
	19. I am satisfied with how well I rested at night.
Care provided after the TAVI procedure	20. Considering my current health, I believe it was worth undergoing the TAVI procedure.
	21. After undergoing the TAVI procedure, I have been able to resume the activities I expected.
	22. I believe that the symptoms I had before the procedure have improved.
	23. I believe that the information provided during the pre-admission visits helped me organize myself and to know how the whole process was going to be.
	24. I am satisfied with the information about the medication I needed to take after discharge.
	25. I believe that the length of my hospital stay for the TAVI procedure was appropriate.
	26. I am satisfied with the follow-up after the TAVI procedure and knowing whom to contact in case of any questions.
	27. I believe that the nurse who accompanied me throughout the process was important for my peace of mind, information, answering questions and support.
Overall Satisfaction with the Care Received	28. Please rate your overall satisfaction with the service you received during the entire process on a scale from 1 to 10.
	29. I would recommend that other patients in my same situation undergo the TAVI procedure.

Abbreviations: TAVI = transcatheter aortic valve implantation.

**Table 2:** Sociodemographic and clinical characteristics of patients by validation phase (*n* = 335).

	1st Evaluation 2023 (*n* = 100)	2nd Evaluation 2024 (*n* = 235)	*p*
Sex (*n*; %)			
Female	55 (55%)	119 (50.6%)	0.465
Male	45 (45%)	116 (49.4%)	
Age (mean; SD)			
Age	81.1 (5.39)	81.1 (5.34)	0.914
Educational level			
No Education	4 (4%)	5 (2.1%)	0.150
Basic Level	62 (62%)	130 (55.3)	
High School	27 (27%)	91 (38.7)	
University	3 (3%)	6 (2.6)	
Other	4 (4%)	3 (1.3)	
Cognitive impairment			
None-normal	86 (86%)	201 (85.5)	0.211
Mild	8 (8%)	22 (9.4%)	
Moderate	3 (3%)	11 (4.7)	
Severe	3 (3%)	1 (0.4)	
Clinical characteristics			
Hypertension	78 (78%)	174 (74.0%)	0.443
Dyslipidaemia	75 (75%)	163 (69.4)	0.298
Diabetes	43 (43%)	73 (31.1)	0.036
Renal impairment	23 (23%)	65 (27.7)	0.375
NYHA			
I	13 (13%)	18 (7.9)	0.120
II	55 (55%)	108 (47.2)	
IIII	26 (26%)	80 (34.9)	
IV	6 (6%)	23 (10.0)	
SD: standard deviation			

**Table 3:** Distribution of frequencies and percentages by item of the VALVEX questionnaire according to phase (2023 y 2024).

Questions: Care Provided Before the TAVI Procedure												
	First Evaluation 2023						Second Evaluation 2024					
		Strongly disagree	Disagree	Neither agree nor disagree	Agree	Strongly agree		Strongly disagree	Disagree	Neither agree nor disagree	Agree	Strongly agree
	*n*	%	%	%	%	%	*n*	%	%	%	%	%
Q1.	100	4.0	4.0	3.0	44.0	45.0	235	3.8	3.8	4.3	40.4	47.7
Q2.	100	4.0	9.0	7.0	38.0	42.0	235	2.6	5.1	6.4	37.0	48.9
Q3.	100	-	-	10.0	23.0	67.0	235	-	1.6	7.7	24.7	66.0
Q4.	100	-	1.0	-	33.0	66.0	235	0.4	1.7	2.1	29.8	66.0
Q5.	95	-	1.1	2.1	24.2	72.6	235	-	0.8	2.6	24.3	72.3
**Questions: Care provided during the TAVI procedure and hospital admission**												
	*n*	Strongly disagree	Disagree	Neither agree nor disagree	Agree	Strongly agree	*n*	Strongly disagree	Disagree	Neither agree nor disagree	Agree	Strongly agree
Q6.	100	1.0	2.0	4.0	34.0	59.0	234	0.4	2.6	4.3	32.9	59.8
Q7.	100	4.0	12.0	10.0	40.0	34.0	234	3.8	8.1	9.4	32.1	46.6
Q8.	100	-	1.0	2.0	30.0	67.0	235	0.4	0.9	2.6	28.5	67.7
Q9.	100	-	3.0	1.0	29.0	67.0	235	-	1.7	2.1	27.7	68.5
Q10.	100	-	-	4.0	28.0	68.0	235	-	0.4	3.0	27.2	69.4
Q11.	100	-	1.0	4.0	35.0	60.0	235	-	1.3	1.7	29.8	67.2
Q12.	100	-	-	2.0	36.0	62.0	235	-	-	2.6	31.0	66.4
Q13.	100	-	1.0	1.0	31.0	67.0	235	-	0.9	2.1	27.2	69.8
Q14.	100	-	3.0	3.0	31.0	63.0	235	-	2.1	1.7	28.1	68.1
**Questions: Facilities**												
	*n*	Strongly disagree	Disagree	Neither agree nor disagree	Agree	Strongly agree	*n*	Strongly disagree	Disagree	Neither agree nor disagree	Agree	Strongly agree
Q15.	100	-	2.0	1.0	42.0	55.0	235	-	2.1	2.6	35.3	60.0
Q16.	100	3.0	4.0	8.0	36.0	49.0	235	2.6	3.4	6.4	33.6	54.0
Q17.	100	5.0	11.0	20.0	38.0	26.0	235	5.1	11.1	18.3	34.5	31.0
Q18.	100	1.0	2.0	10.0	39.0	48.0	235	0.9	3.8	7.2	34.9	53.2
Q19.	100	1.0	4.0	6.0	42.0	47.0	234	0.9	3.8	7.3	35.9	52.1
**Questions: Care provided after the TAVI procedure**												
	*n*	Strongly disagree	Disagree	Neither agree nor disagree	Agree	Strongly agree	*n*	Strongly disagree	Disagree	Neither agree nor disagree	Agree	Strongly agree
Q20.	100	1.0	1.0	9.0	19.0	70.0	235	0.4	3.1	8.5	19.1	68.9
Q21.	100	2.0	4.0	18.0	25.0	51.0	235	3.4	5.1	16.6	23.0	51.9
Q22.	99	-	4.1	12.1	20.2	63.6	235	2.1	3.4	11.9	20.5	62.1
Q23.	99	2.0	1.0	9.1	22.2	65.7	234	1.3	1.7	10.3	25.6	61.1
Q24.	100	1.0	4.0	6.0	25.0	64.0	235	0.9	2.6	3.8	25.5	67.2
Q25.	100	-	1.0	10.0	30.0	59.0	235	0.4	2.1	8.9	26.0	62.6
Q26.	100	-	-	13.0	26.0	61.0	235	-	-	11.5	25.1	63.4
Q27.	95	-	1.1	6.3	22.1	70.5	235	-	0.4	6.4	20.9	71.9

Abbreviations: Q = question; TAVI = Transcatheter Aortic Valve Implantation.

**Table 4:** Overall distribution of frequencies and percentages by item of the VALVEX questionnaire (*n* = 335).

Questions: Care Provided Before the TAVI Procedure						
	*n*	Strongly disagree	Disagree	Neither agree nor disagree	Agree	Strongly agree
		%	%	%	%	%
Q1.	335	3.9	3.9	3.9	41.5	46.9
Q2.	335	3.0	6.3	6.6	37.3	46.9
Q3.	335	-	1.2	8.4	24.2	66.3
Q4.	335	0.3	1.5	1.5	30.7	66.0
Q5.	330	-	0.6	2.4	24.2	72.4
**Questions: Care provided during the TAVI procedure and hospital admission**						
	*n*	Strongly disagree	Disagree	Neither agree nor disagree	Agree	Strongly agree
Q6.	334	0.6	2.4	4.2	33.2	59.6
Q7.	334	3.9	9.3	9.6	34.4	42.8
Q8.	335	0.3	0.9	2.4	29.0	67.5
Q9.	335	-	2.1	1.8	28.1	68.1
Q10.	335	-	0.3	3.3	27.5	69.0
Q11.	335	-	1.2	2.4	31.3	65.1
Q12.	335	-	-	2.4	32.5	65.1
Q13.	335	-	0.9	1.8	28.4	69.0
Q14.	335	-	2.4	2.1	29.0	66.6
**Questions: Facilities**						
	*n*	Strongly disagree	Disagree	Neither agree nor disagree	Agree	Strongly agree
Q15.	335	-	2.1	2.1	37.3	58.5
Q16.	335	2.7	3.6	6.9	34.3	52.5
Q17.	335	5.1	11.0	18.8	35.5	29.6
Q18.	335	0.9	3.3	8.1	36.1	51.6
Q19.	334	0.9	3.9	6.9	37.7	50.6
**Questions: Care provided after the TAVI procedure**						
	*n*	Strongly disagree	Disagree	Neither agree nor disagree	Agree	Strongly agree
Q20.	335	0.6	2.4	8.7	19.1	69.3
Q21.	335	3.0	4.8	17.0	23.6	51.6
Q22.	334	1.5	3.6	12.0	20.4	62.6
Q23.	333	1.5	1.5	9.9	24.6	62.5
Q24.	335	0.9	3.0	4.5	25.4	66.3
Q25.	335	0.3	1.8	9.3	27.2	61.5
Q26.	335	-	-	11.9	25.4	62.7
Q27.	330	-	0.6	6.4	21.2	74.5

Abbreviations: Q = question; TAVI = Transcatheter Aortic Valve Implantation.

**Table 5:** Internal consistency by dimension, phase, and overall consistency of the VALVEX questionnaire.

	1st Evaluation				2nd Evaluation				Global			
VALVEX Questionnaire	*n*	Mean	SD	Cronbach’s Alpha	*n*	Mean	SD	Cronbach’s Alpha	*n*	Mean	SD	Cronbach’s Alpha
**Pre-procedure section**												
Questions 1–5	95	22.12	2.5	0.695	235	22.3	2.7	0.723	330	22.25	2.66	0.713
**Intra-procedure section**												
Questions 6–14	100	40.55	4.3	0.864	234	41.03	4.6	0.905	334	40.89	4.557	0.893
**Facilities section**												
Questions 15–19	100	21.04	3.3	0.811	234	21.32	3.200	0.760	334	21.23	3.274	0.776
**Post-procedure/Follow-up section**												
Questions 20–29	94	54.12	6.3	0.879	234	54.22	7.5	0.761	328	54.19	7.218	0.787

Abbreviations: SD = standard deviation.

## Data Availability

Data are available upon request from the authors. The raw data supporting the conclusions of this article will be made available by the authors on request.

## Abbreviations

AS	aortic stenosis
EACTS	European association for cardio-thoracic surgery
ICC	intraclass correlation coefficient
PREMS	patient reported experience measure
PROMS	patient reported outcomes measure
SEC	Spanish society of cardiology
TAVI	transcatheter aortic valve implantation

## Appendix A

**Table A1:** Valvex questionnaire on patient experience for transcatheter valve implant.

ID number: …………	Sex	□ Male		Age: …………	
		□ Female			
**Care provided before the TAVI procedure**	Strongly disagree	Disagree	Neither agree nor disagree	Agree	Strongly agree
1. When I was diagnosed with the disease, they explained to me other treatment options (if there were any)	□	□	□	□	□
2. When I was diagnosed with the disease, I was able to take part in the decision to undergo TAVI.	□	□	□	□	□
3. I believe that the decision to undergo the TAVI procedure was the right one.	□	□	□	□	□
4. I am satisfied with the information provided by the medical staff when I was diagnosed with my disease.	□	□	□	□	□
5. I am satisfied with the information provided by the nursing staff when I was diagnosed with my disease.	□	□	□	□	□
**Care provided during the TAVI procedure and hospital admission**	Strongly disagree	Disagree	Neither agree nor disagree	Agree	Strongly agree
6. I believe it was easy to get to the unit where I had to be admitted.	□	□	□	□	□
7. I believe that the waiting time from diagnosis to treatment (TAVI procedure) was appropriate.	□	□	□	□	□
8. I am satisfied with the care received during the procedure.	□	□	□	□	□
9. I am satisfied with the information the doctors gave me throughout my hospital stay.	□	□	□	□	□
10. I am satisfied with the information the nursing staff in the units provided me throughout my hospital stay.	□	□	□	□	□
11. I am satisfied with the kindness and professionalism with which the healthcare staff treated me.	□	□	□	□	□
12. I believe that the healthcare staff explained the process to me in easy-to-understand language.	□	□	□	□	□
13. I believe that the time spent by the unit nurse was adequate.	□	□	□	□	□
14. I am satisfied with the care provided by the healthcare staff during my stay.	□	□	□	□	□
**Facilities**	Strongly disagree	Disagree	Neither agree nor disagree	Agree	Strongly agree
15. I am satisfied with the cleanliness of the facilities.	□	□	□	□	□
16. I am satisfied with the noise level in the facilities.	□	□	□	□	□
17. I am satisfied with the meals that were served to me.	□	□	□	□	□
18. I am satisfied with the comfort of the room.	□	□	□	□	□
19. I am satisfied with how well I rested at night.	□	□	□	□	□
**Care provided after the TAVI procedure**	Strongly disagree	Disagree	Neither agree nor disagree	Agree	Strongly agree
20. Considering my current health, I believe it was worth undergoing the TAVI procedure.	□	□	□	□	□
21. After undergoing the TAVI procedure, I have been able to resume the activities I expected.	□	□	□	□	□
22. I believe that the symptoms I had before the procedure have improved.	□	□	□	□	□
23. I believe that the information provided during the pre-admission visits helped me organize myself and to know how the whole process was going to be.	□	□	□	□	□
24. I am satisfied with the information about the medication I needed to take after discharge.	□	□	□	□	□
25. I believe that the length of my hospital stay for the TAVI procedure was appropriate.	□	□	□	□	□
26. I am satisfied with the follow-up after the TAVI procedure and knowing whom to contact in case of any questions.	□	□	□	□	□
27. I believe that the nurse who accompanied me throughout the process was important for my peace of mind, information, answering questions, and support.	□	□	□	□	□
**Overall satisfaction with the care received**	1 2	3 4	5 6	7 8	9 10
28. Please rate your overall satisfaction with the service you received during the entire process on a scale from 1 to 10.	□ □	□ □	□ □	□ □	□ □
29. I would recommend that other patients in my same situation undergo the TAVI procedure.	□ □	□ □	□ □	□ □	□ □

Abbreviations: TAVI = Transcatheter Aortic Valve Implantation.
